# Unveiling the causal effects of gut microbiome on trimethylamine N-oxide: evidence from Mendelian randomization

**DOI:** 10.3389/fmicb.2024.1465455

**Published:** 2024-10-25

**Authors:** Yunfeng Yu, Yuman Yin, Juan Deng, Xinyu Yang, Siyang Bai, Rong Yu

**Affiliations:** ^1^School of Traditional Chinese Medicine, Hunan University of Chinese Medicine, Changsha, Hunan, China; ^2^Department of Endocrinology, The First Hospital of Hunan University of Chinese Medicine, Changsha, Hunan, China

**Keywords:** gut microbiome, trimethylamine N-oxide, relationship, genome-wide association studies, Mendelian randomization

## Abstract

**Objective:**

The relationship between gut microbiome and trimethylamine oxide (TMAO) has not been fully elucidated. We aimed to assess the causal effects of different gut microbes on TMAO using Mendelian randomization (MR).

**Methods:**

Gut microbiome and TMAO datasets were acquired from genome-wide association studies and screened for single nucleotide polymorphisms according to the basic assumptions of MR. Inverse variance weighted was used as the main method in MR analysis to assess the causal relationship between the gut microbiome and TMAO. Finally, the MR-Egger intercept, Cochran's Q test, and leave-one-out sensitivity analysis were used to assess the horizontal pleiotropy, heterogeneity, and robustness of the results, respectively.

**Results:**

MR analysis revealed that the *species Bacteroides finegoldii* (odds ratio [OR] 1.064, 95% confidence interval [CI] 1.003 to 1.128, *p* = 0.039), *family Sutterellaceae* (OR 1.188, 95% CI 1.003 to 1.407, *p* = 0.047), and *phylum Pseudomonadota* (OR 1.205, 95% CI 1.036 to 1.401, *p* = 0.016), as well as the *species Bacteroides uniformis* (OR 1.263, 95% CI 1.039 to 1.535, *p* = 0.019), were positively associated with increased genetic susceptibility to TMAO. In contrast, the *species Bacteroides thetaiotaomicron* (OR 0.813, 95% CI 0.696 to 0.950, *p* = 0.009) and *Bilophila wadsworthia* (OR 0.828, 95% CI 0.690 to 0.995, *p* = 0.044) were associated with reduced genetic susceptibility to TMAO. Additionally, the MR-Egger intercept indicated no horizontal pleiotropy (*p* ≥ 0.05), and Cochran's Q test and sensitivity analysis demonstrated that the results were not heterogeneous (*p* ≥ 0.05) and were robust.

**Conclusion:**

Our findings revealed the role of the *phylum Pseudomonadota, family Sutterellaceae, species Bacteroides finegoldii*, and *Bacteroides uniformis* in increasing TMAO, as well as the *species Bacteroides thetaiotaomicron* and *Bilophila wadsworthia* in decreasing TMAO. This study provides new insights into the relationship between the gut microbiome and TMAO levels.

## 1 Introduction

Gut microbiome is the microbial community in the gut composed of bacteria, fungi, archaea, prokaryotes, eukaryotes, and viruses (Hillman et al., 2017). It is associated with the health of its host organism and plays a role in the digestion, absorption, metabolism, and synthesis of vitamins as well as the regulation of immune function (Tzeng and Lee, 2024; Dong et al., 2024). The gut microbiome is predominantly composed of bacteria, with the most common phyla being *Firmicutes, Bacteroidetes, Pseudomonadota*, and *Fusobacteria* (Hillman et al., 2017). Among them, the *phyla Firmicutes* and *Bacteroidetes* account for more than 90% of the total number of gut microbe, and their ratio serves as a biological marker for several diseases (Yañez et al., 2021). Previous studies have shown that gut microbes are associated with many diseases such as cardiovascular diseases, neurodegenerative diseases, diabetes, and osteoporosis (Xie et al., 2024; Andreu-Sánchez et al., 2024). Subsequent studies have found that the mechanisms by which gut microbes influence diseases may be related to the regulation of metabolites such as trimethylamine N-oxide (TMAO) (Li et al., 2019, 2022). The metabolites represented by TMAO may be essential mediators of gut microbes that influence the health of organisms.

TMAO is a metabolite of gut microbes, whose synthesis is influenced by factors such as diet, intestinal microbiota, and the activity of flavin-containing monooxygenase 3 (FMO3) (Zhang et al., 2023). Trimethylamine (TMA), a precursor of TMAO, is generated from choline, phosphatidylcholine, and carnitine through metabolism by intestinal microbes (Badaoui et al., 2024). Subsequently, TMA enters blood circulation and is oxidized in the liver by FMO3 to generate TMAO (Zhang et al., 2023). As research has progressed, TMAO is associated with diseases such as cardiovascular disease, kidney disease, diabetes, cancer, and Alzheimer's disease and is increasingly recognized as the link between gut microbes and diseases (Subramaniam and Fletcher, 2018; Wang et al., 2023). Subsequent studies have pointed out that TMAO induces disease through the activation of NF-κB-mediated inflammatory responses and is considered an important metabolite associated with disease (Zhen et al., 2023; Constantino-Jonapa et al., 2023). However, < 1% of the vast number of gut microbes possess genes required to produce TMAO (Rath et al., 2017). Although previous studies have reported that several intestinal microbiota are involved in its synthesis (Zhang and Jian, 2023), the relationship between different gut microbes and TMAO has not been comprehensively assessed. Additionally, limited by the nature of the study, the causal attributes of different gut microbes and TMAO require further clarification. A novel and effective method is needed to comprehensively assess the causal effects of the gut microbiome on TMAO.

Mendelian randomization (MR) is a cutting-edge epidemiological research method based on genetic epidemiology (Smith and Ebrahim, 2003). It uses genetic variants as instrumental variables to assess the causal effects of exposure on outcomes, effectively overcoming the limitations of traditional observational studies (Birney, 2022). As MR follows the rule of random assignment, it is less susceptible to confounding variables and reverse causality than traditional cross-sectional and observational studies (Birney, 2022). Additionally, because of its vast dataset, MR can provide a more comprehensive analysis of causal effects. In this study, we assessed the relationship between different gut microbes and TMAO using two-sample MR.

## 2 Materials and methods

### 2.1 Study design

MR stands out as a robust approach for evaluating causal relationships. It serves as a valuable analytical technique when experimental data are limited and is particularly effective in addressing confounding and reverse causality concerns within observational data. Its strength lies in its ability to simulate the control of randomization over the experimental design, thus reducing possible confounding variables and biases as well as improving the accuracy of causal inferences. When experimental evidence is sufficient and the results are not reverse causal or confounding, MR analysis is no longer of sufficient value.

**Figure 1:**
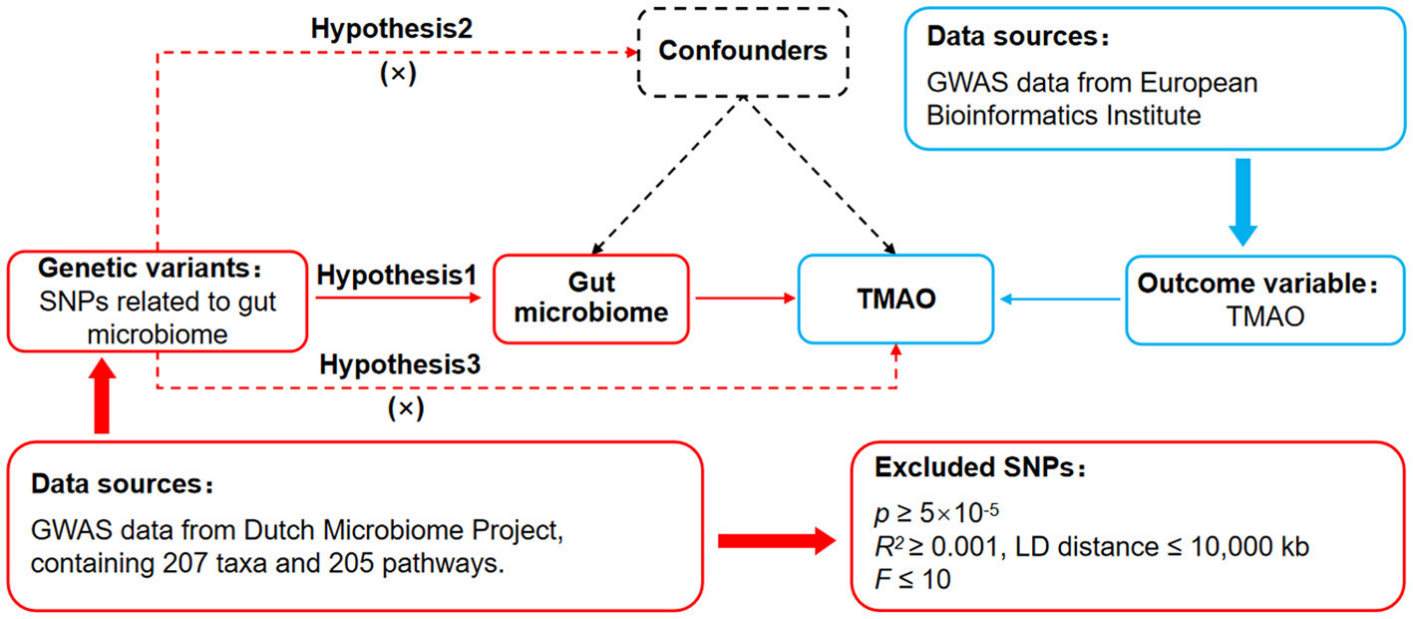
MR design to analyze the effects of the gut microbiome on genetic susceptibility to TMAO. MR, Mendelian randomization; TMAO, trimethylamine N-oxide.

The causal effects of different gut microbiomes on TMAO have not been fully elucidated; therefore, we employed MR analysis to explore the gut microbiomes that have a significant effect on TMAO. The principle of MR is to analyze the causal effect of exposure on outcomes through single nucleotide polymorphisms (SNPs), which are common variations in the genome. This requires the MR design to meet three basic assumptions (Davies et al., 2018) (Figure 1): assumption 1 (association assumption) is that SNPs are strongly associated with radiation exposure, assumption 2 (independence assumption) is that SNPs are independent of confounding variables, and assumption 3 (exclusivity assumption) is that SNPs cannot affect outcomes through pathways other than exposure. Based on these assumptions, the MR analysis identified SNPs that were significantly associated with each gut microbiota. We then assessed the independent impact of these SNPs on TMAO to uncover certain microbiota within the gut microbiome dataset that had a notable influence on TMAO.

### 2.2 Data sources

Gut microbiome data were obtained from the Dutch Microbiome Project (Lopera-Maya et al., 2022). Shotgun metagenomic sequencing was used to report genome-wide association study (GWAS) data for 207 taxa and 205 pathways by testing the feces of 7,738 Europeans. The data were derived from a secondary analysis of two longitudinal cohort data by Panyard et al. (2021) and were included in the European Bioinformatics Institute. They performed metabolomic analysis on 291 individuals of European ancestry by ultrahigh-performance liquid chromatography-tandem mass spectrometry and finalized 338 metabolites, including TMAO, referenced as ebi-a-GCST90026279.

### 2.3 Genetic tool variable selection

First, qualifying *p* < 5 × 10^−5^, SNPs that strongly correlated with the gut microbiome were searched to satisfy Assumption 1. Qualifying *R*^2^ < 0.001 and kb = 10,000, we then searched for independent SNPs to exclude interference from linkage disequilibrium. *F* > 10 was then limited to searching for strongly correlated SNPs to exclude interference from weakly correlated variables. *F* was calculated as *F* = [*R*^2^/(1-*R*^2^)] ^*^ [(*N*-*K*-1)/*K*], where *K* refers to the number of paired samples, *N* is the total number of samples, and *R*^2^ is the cumulative explained variance. Afterward, SNPs containing confounding variables were excluded using PhenoScanner to satisfy Assumption 2. Palindromic and ambiguous SNPs were then excluded based on the effect of allele frequency when adjusting the allele orientation for exposure and outcome. Finally, the MR-Pleiotropy RESidual Sum and Outlier were used to exclude aberrant SNPs (*p* < 1) to ensure the correctness of the causal inference.

### 2.4 Data analysis

This study followed the STROBE-MR guidelines (Skrivankova et al., 2021). The “TwoSampleMR (0.5.7)” program package for R 4.3.1 was used to conduct a two-sample MR analysis of the gut microbiome with TMAO. Inverse variance weighted (IVW) was set as the primary tool for the MR analysis, whereas the MR-Egger, weighted mode, weighted median, and simple mode were set as secondary tools. IVW is typically used as the primary tool for MR analysis when the assumptions of instrument strength and the absence of pleiotropy hold. Additionally, MR-Egger and weighted mode are worth referring to when the results have pleiotropy; the weighted median is beneficial when there is potential heterogeneity in genetic instruments affecting the analysis results, and the simple mode is a commonly used secondary tool that does not consider horizontal pleiotropy or other potential complexities.

Subsequently, the MR-Egger intercept was used to evaluate horizontal pleiotropy, with *p* ≥ 0.05 indicating that the results had no horizontal pleiotropy to satisfy Assumption 3. Cochran's Q was used to assess heterogeneity, with *p* ≥ 0.05 indicating no significant heterogeneity of the results. Finally, a leave-one-out sensitivity analysis was used to assess the robustness of the results. The lack of significant changes in the pooled effect size indicated that the results were robust.

## 3 Results

### 3.1 Genetic instrumental variables

After testing the assumptions of association, independence, and exclusivity, MR analysis revealed that six gut microbes were significantly associated with genetic susceptibility to TMAO. Among them, 11 SNPs for the *phylum Pseudomonadota*, nine SNPs for the *family Sutterellaceae*, seven for the *species Bilophila wadsworthia*, 18 for the *species Bacteroides finegoldii*, eight for the *species Bacteroides uniformis*, and eight SNPs for the *species Bacteroides thetaiotaomicron* were included in the MR analysis (Supplementary Table S1).

### 3.2 Two-sample MR analysis

**Figure 2:**
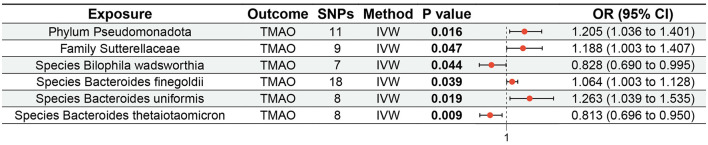
Forest plot of MR analysis for the effects of the gut microbiome on genetic susceptibility to TMAO. MR, Mendelian randomization; TMAO, trimethylamine N-oxide.

**Figure 3:**
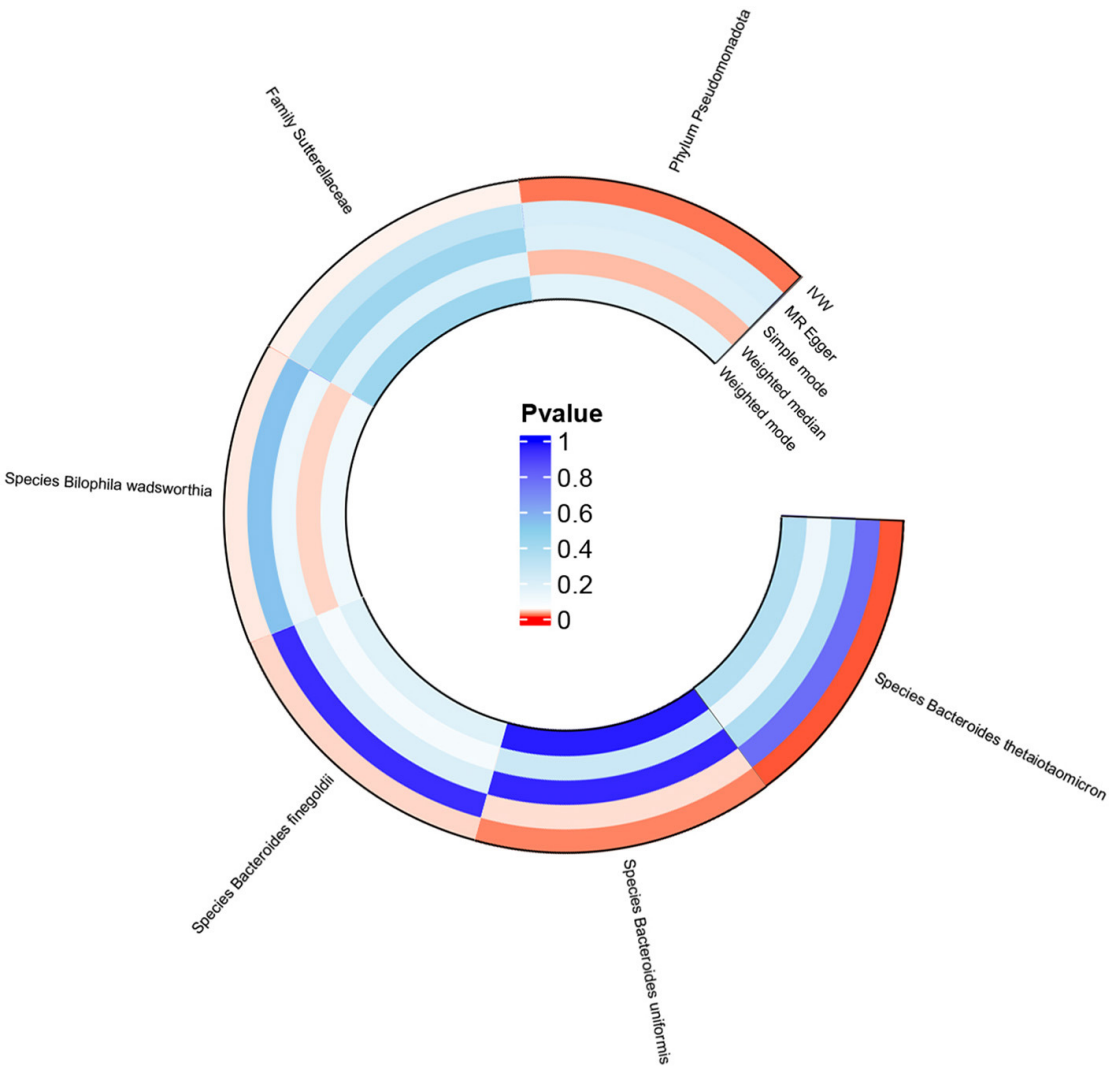
Circle plot of MR analysis for the effects of the gut microbiome on genetic susceptibility to TMAO. MR, Mendelian randomization; TMAO, trimethylamine N-oxide.

**Figure 4:**
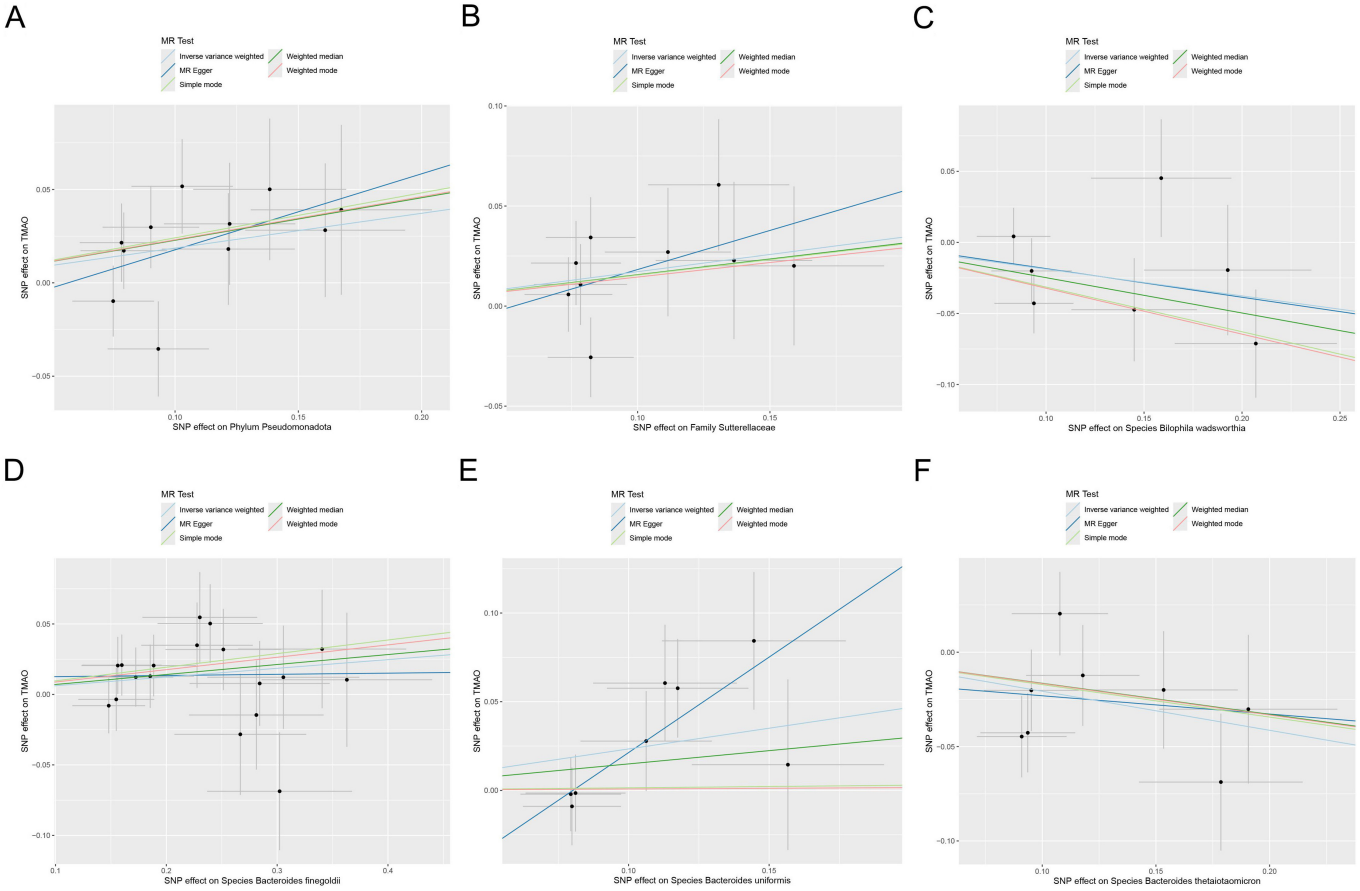
Scatter plot of MR analysis for the effects of the gut microbiome on genetic susceptibility to TMAO. **(A)** *Phylum Pseudomonadota*; **(B)** *family Sutterellaceae*; **(C)** *species Bilophila wadsworthia*; **(D)** *species Bacteroides finegoldii*; **(E)** *species Bacteroides uniformis*; **(F)** *species Bacteroides thetaiotaomicron*. MR, Mendelian randomization; TMAO, trimethylamine N-oxide.

MR analysis based on IVW revealed that the *species Bacteroides finegoldii* (odds ratio [OR] 1.064, 95% confidence interval [CI] 1.003 to 1.128, *p* = 0.039), *family Sutterellaceae* (OR 1.188, 95% CI 1.003 to 1.407, *p* = 0.047), and *phylum Pseudomonadota* (OR 1.205, 95% CI 1.036 to 1.401, *p* = 0.016), as well as the *species Bacteroides uniformis* (OR 1.263, 95% CI 1.039 to 1.535, *p* = 0.019), were positively associated with increased genetic susceptibility to TMAO. In contrast, the *species Bacteroides thetaiotaomicron* (OR 0.813, 95% CI 0.696 to 0.950, *p* = 0.009) and *Bilophila wadsworthia* (OR 0.828, 95% CI 0.690 to 0.995, *p* = 0.044) were associated with reduced genetic susceptibility to TMAO (Figure 2). Figure 3 summarizes the causal effects of the gut microbiome on TMAO obtained using the five methods, and Figure 4 shows the independent effects of each SNP. The MR-Egger intercept indicated no horizontal pleiotropy (*p* ≥ 0.05) (Supplementary Table S2).

### 3.3 Heterogeneity and sensitivity analysis

**Figure 5:**
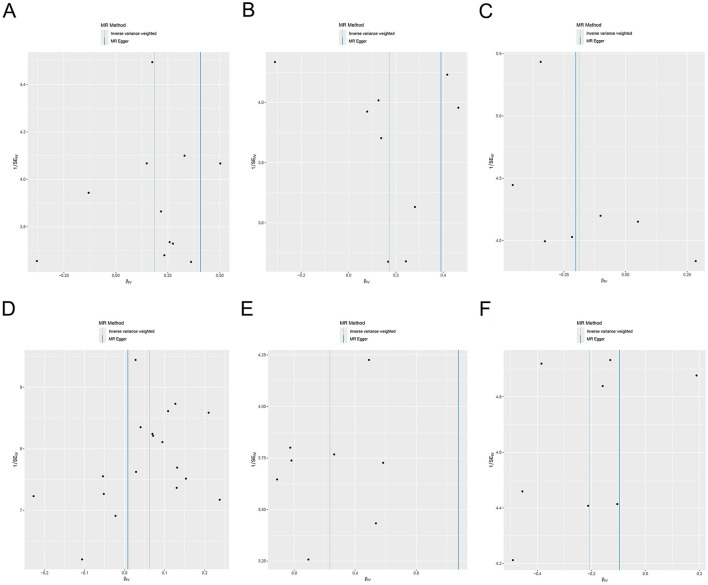
Funnel plot of heterogeneity analysis for the effects of the gut microbiome on genetic susceptibility to TMAO. **(A)** *Phylum Pseudomonadota*; **(B)** *family Sutterellaceae*; **(C)** *species Bilophila wadsworthia*; **(D)** *species Bacteroides finegoldii*; **(E)** *species Bacteroides uniformis*; **(F)** *species Bacteroides thetaiotaomicron*. TMAO, trimethylamine N-oxide.

**Figure 6:**
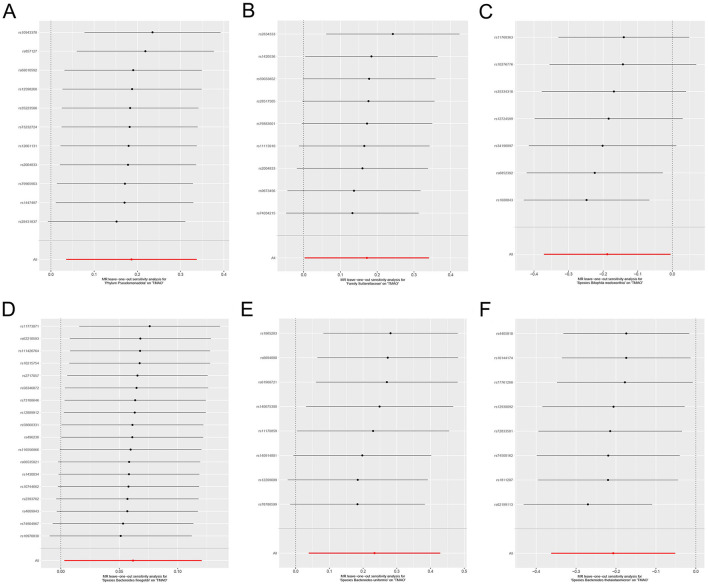
Leave-one-out sensitivity analysis for the effects of the gut microbiome on genetic susceptibility to TMAO. **(A)** *Phylum Pseudomonadota*; **(B)** *family Sutterellaceae*; **(C)** *species Bilophila wadsworthia*; **(D)** *species Bacteroides finegoldii*; **(E)** *species Bacteroides uniformis*; **(F)** *species Bacteroides thetaiotaomicron*. TMAO, trimethylamine N-oxide.

Cochran's Q demonstrated no significant heterogeneity (*p* ≥ 0.05) in the results (Supplementary Table S3, Figure 5). The leave-one-out sensitivity analysis revealed that the MR results were robust (Figure 6).

## 4 Discussion

### 4.1 Research background and results

**Figure 7:**
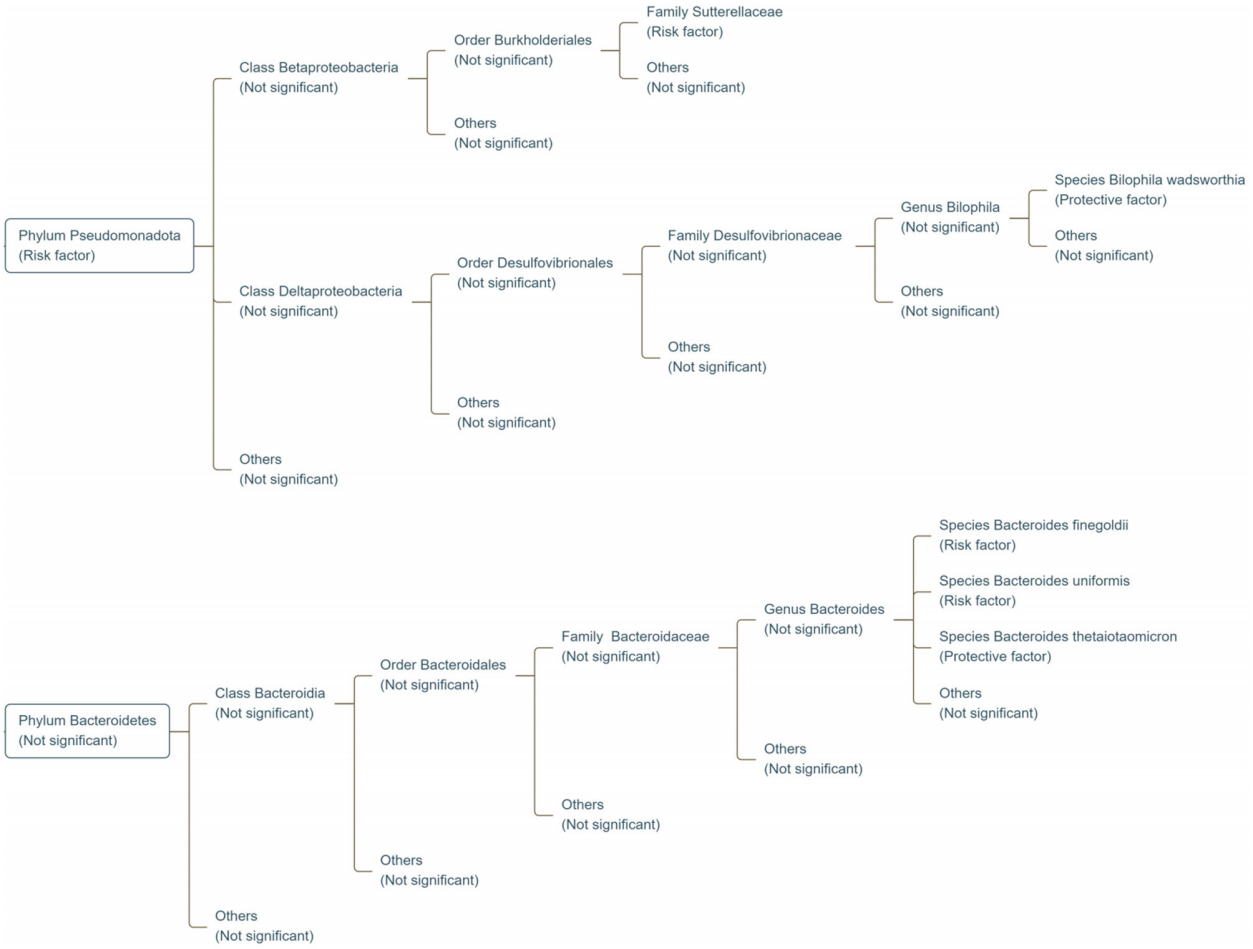
Classification map of gut microbiota significantly associated with genetic susceptibility to TMAO. Risk factor refers to the factors associated with increased genetic susceptibility to TMAO; protective factor refers to those associated with reduced genetic susceptibility to TMAO; not significant refers to no significant relationship with TMAO.

TMAO, a significant metabolite produced by gut microbes, has critical implications in the development of many diseases (Spasova et al., 2024). Although previous studies have provided preliminary insights into the relationship between gut microbes and TMAO (Romano et al., 2015), they are insufficient to adequately explain causal effects (Jin et al., 2024). To the best of our knowledge, this is the first MR analysis to assess the causal relationship between the gut microbiome and TMAO using GWAS. Our findings revealed the role of *Bacteroides finegoldii, Bacteroides uniformis, Bacteroides thetaiotaomicron*, and *Bilophila wadsworthia* at the species level, *Sutterellaceae* at the family level, and *Pseudomonadota* (formerly *Proteobacteria*) at the phylum level in modulating TMAO levels (Figure 7). As these gut microbiota belong to the *phyla Pseudomonadota* and *Bacteroidetes*, MR analysis revealed the potential effects of these phyla on TMAO levels in Europeans. A previous genetic study reported contrasting results to ours (Andreu-Sánchez et al., 2024), in which they found that the *phyla Pseudomonadota* and *Bacteroidetes* were not associated with TMAO levels. We speculate that this discrepancy may stem from different datasets, as the dataset used for this MR analysis was from the Dutch Microbiome Project, whereas the dataset used for the previous genetic study was from the Rotterdam Study III-2 and LifeLines-DEEP (Andreu-Sánchez et al., 2024). Additionally, although previous studies support that the *phyla Pseudomonadota* and *Bacteroidetes* affect the prognosis of cardiovascular disease by increasing TMAO levels (Martins et al., 2024; Melnychuk and Lizogub, 2022), the MR analysis by Wang et al. (2023) did not find a significant association between these phyla and cardiovascular disease. We speculate that this may be because this previous study concentrated solely on species with consistent positive findings across multiple databases, leading to a focus on the role of *genus RuminococcusUCG010* in cardiovascular disease.

### 4.2 Effects of *phylum Pseudomonadota* on TMAO

*Pseudomonadota* is one of the most abundant and diverse phyla in the bacterial community, containing several common beneficial and pathogenic bacteria such as the *species Escherichia coli, Serratia marcescens*, and *Helicobacter pylori* (Rizzatti et al., 2017). A clinical study conducted in China revealed that the abundance of *phylum Pseudomonadota* was significantly higher in patients with preeclampsia (4.51%) than in healthy controls (2.56%) (Wang et al., 2019). Similarly, the plasma concentration of TMAO was higher in preeclampsia patients (2.17 ± 1.35 μmol/L) compared to controls (1.36 ± 0.72 μmol/L) (Wang et al., 2019). A meta-analysis involving 67 studies also found a potential association between the *phylum Pseudomonadota* and serum TMAO, which demonstrated that cardiovascular patients had significantly higher levels of both the *phylum Pseudomonadota* (standardized mean difference [SMD]: 0.33, 95% CI: 0.18–0.49) and plasma TMAO than healthy controls (SMD: 0.42, 95% CI: 0.17–0.66) (Martins et al., 2024). Another study in China found that a daily calorie-restricted diet increased the relative abundance of the *phylum Pseudomonadota* by 3.39 times and dramatically increased circulating TMAO in mice compared to normal feeding (Zhang et al., 2022). This evidence suggests that the abundance of the *phylum Pseudomonadota* may be positively correlated with the serum TMAO concentration. In addition, subsequent studies have indicated that the *phylum Pseudomonadota* may increase the synthesis of TMAO by regulating CutC (Huang et al., 2024). CutC is a choline TMA lyase commonly found in choline-fermenting bacteria that converts choline into the TMAO precursor TMA (Martínez-del Campo et al., 2015). A Chinese animal study indicated that a methionine-restricted diet significantly inhibited the growth of TMA-producing bacteria in the *phylum Pseudomonadota* by downregulating CutC expression, which in turn reduced TMAO levels in mice fed a high-fat diet (Lu et al., 2023). In summary, the *phylum Pseudomonadota* may synthesize TMA to increase TMAO levels, and its synthesis is regulated by CutC. This suggests that the abundance of the *phylum Pseudomonadota* may be positively correlated with serum TMAO concentrations.

Additionally, this MR study revealed that the *family Sutterellaceae* within *phylum Pseudomonadota* is associated with increased TMAO levels. *Family Sutterellaceae* is a family of Gram-negative bacteria that includes genera such as *Sutterella, Mesosutterella, Parasutterella*, and *Turicimonas*. A clinical study in Korea found that patients with polycystic ovary syndrome showed a significant increase in the abundance of the intestinal *family Sutterellaceae*, which led to elevated circulating TMAO levels and promoted the progression of cardiovascular disease, diabetes mellitus, and non-alcoholic fatty liver disease (Singh et al., 2024). Another animal experiment in China indicated that the relative abundance of *genus Sutterella* and circulating TMAO were significantly elevated in diabetic rats with erectile dysfunction compared to healthy rats (Li et al., 2017). These findings support a positive correlation between TMAO and the abundance of *family Sutterellaceae*. In addition, we speculate that the *family Sutterellaceae* may elevate TMAO levels by promoting TMA synthesis.

Our findings also indicate that the *species Bilophila wadsworthia*, which belongs to the *phylum Pseudomonadota*, is associated with decreased TMAO levels. *Species Bilophila wadsworthia* is a species belonging to the *phylum Pseudomonadota family Desulfovibrionaceae genus Bilophila*, and is the most studied and well-documented model species of *genus Bilophila*. Unfortunately, there is currently no literature documenting a direct relationship between the *species Bilophila wadsworthia* and TMAO, though the available literature suggests that the *Bilophila* genus is associated with decreased TMAO. A study in the United States suggested that the *genus Bilophila* reduced TMAO synthesis by converting TMA to dimethylamine (DMA) (Kivenson and Giovannoni, 2020). It has also been found that the *genus Bilophila* reduces cardiovascular disease by lowering circulating TMAO levels (Kivenson and Giovannoni, 2020), supporting TMAO as a link between gut microbes and disease. Another study in Sweden reported that the abundance of *genus Bilophila* was associated with reduced blood lipid levels and cardiovascular risk markers (Marungruang et al., 2018); this effect may have been achieved by modulating TMAO. These findings indicate that the *genus Bilophila* inhibits the synthesis of TMAO by converting TMA to DMA, suggesting that the *species Bilophila wadsworthia* may also be associated with reduced TMAO.

### 4.3 Effects of *phylum Bacteroidetes* on TMAO

*Phylum Bacteroidetes* consists of Gram-negative bacteria that are non-endospore, anaerobic, aerobic, or rod-shaped, and is the most abundant Gram-negative phylum residing in the gastrointestinal tract (Gibiino et al., 2018; Pan et al., 2023). A clinical study conducted in China revealed that, compared to healthy controls, patients with acute myocardial infarction exhibited significantly elevated levels of *phylum Bacteroidetes* in their gut as well as increased TMAO levels (Qian et al., 2022). Another study in Ukraine demonstrated that the intestinal abundance of *phylum Bacteroidetes* and plasma TMAO levels were significantly increased in atherosclerotic patients with arrhythmia compared to those without (Melnychuk and Lizogub, 2022). Subsequently, an animal experiment in China revealed that compared with normal controls, mice in the diarrhea model group had a significantly increased abundance of *phylum Bacteroidetes* and significantly higher levels of CutC activity as well as TMAO in the cecum of mice (Guo et al., 2024). CutC is a choline TMA lyase whose main function is to convert choline to TMA. It indicates that the *phylum Bacteroidetes* may increase TMA levels by enhancing CutC activity, thereby providing raw materials for TMAO synthesis.

This MR also revealed that the *species Bacteroides finegoldii* and *Bacteroides uniformis*, belonging to the *phylum Bacteroidetes*, were associated with increased levels of TMAO. *Species Bacteroides finegoldii* and *Bacteroides uniformis* are Gram-negative, rod-shaped, obligate anaerobic bacteria. Previous studies have suggested that the *species Bacteroides uniformis* plays an important role in lipid metabolism. A clinical study in China demonstrated that the *species Bacteroides uniformis* reduced the risk of hepatic steatosis and hyperlipidemia in mice, and its abundance was negatively correlated with low density lipoprotein cholesterol (LDL-C) levels (Yan et al., 2022). Another clinical study in China noted that carnitine (a TMAO precursor) levels were negatively correlated with LDL-C levels (Xiong et al., 2022). This implies that the *species Bacteroides uniformis* may increase TMAO levels by promoting carnitine synthesis, which, in turn, reduces LDL-C levels. Subsequently, a clinical study in the Netherlands noted that the *species Bacteroides uniformis* was positively correlated with levels of betaine, another TMAO precursor (*R* = 0.59, *p* = 0.04) (Hartstra et al., 2020). This suggests that the *species Bacteroides uniformis* may increase TMAO levels by promoting the synthesis of carnitine and betaine. However, currently no studies report the relationship between the *species Bacteroides finegoldii* and TMAO; this relationship requires future validation. We did not find any other *Bacteroidetes* species that were associated with increased TMAO levels. From this, we speculate that the effect of *phylum Bacteroides* on TMAO levels may be mediated specifically by the *species Bacteroides finegoldii* and *Bacteroides uniformis*; however, further studies are needed to explore and elucidate the effects of different *phylum Bacteroides* species on TMAO levels.

*Species Bacteroides thetaiotaomicron* is a polysaccharide-degrading bacterium also belonging to the *phylum Bacteroides*. Our MR demonstrated that the *species Bacteroides finegoldii* and *Bacteroides uniformis* were associated with increased TMAO. In contrast, the *species Bacteroides thetaiotaomicron* was associated with reduced TMAO. A longitudinal clinical study in the United States revealed that a healthy plant-based diet is positively associated with the relative abundance of *species Bacteroides thetaiotaomicron* and negatively associated with TMAO (Shen et al., 2024). Although this study did not directly confirm that reduced TMAO was caused by the *species Bacteroides thetaiotaomicron*, it suggests a potential relationship between the two. We speculate that a healthy plant-based diet may reduce TMAO by decreasing the intake of TMA precursor substances and increasing their consumption by bacteria. However, the causal effects of the *phylum Bacteroides* and its subspecies on TMAO need to be further explored.

### 4.4 Implications for clinical applications

The intricate interplay between specific gut microbes and TMAO susceptibility revealed in this study highlights potential interventions to modulate TMAO-related health outcomes and offers significant implications for clinical applications and therapeutic approaches. *Phylum Pseudomonadota, family Sutterellaceae*, and *species Bacteroides finegoldii* were associated with increased susceptibility to TMAO. Based on the results of previous studies, we hypothesize that these gut microbes increase the risk of cardiovascular disease by increasing TMAO levels, and may serve as key targets for personalized therapy aimed at reducing the cardiovascular risks associated with elevated TMAO levels. Conversely, microbial species such as *Bacteroides thetaiotaomicron* and *Bilophila wadsworthia*, which reduce genetic susceptibility to TMAO, suggest promising avenues for therapeutic interventions that may help lower TMAO levels and potentially improve overall health outcomes. In light of these findings, future research and clinical endeavors should explore the development of tailored probiotic or prebiotic interventions to effectively modulate gut microbiome composition and target TMAO-related pathways with precision, offering novel therapeutic strategies for managing TMAO-associated conditions. This study elucidated the practical implications of these microbial relationships and their implications for future interventions, setting the stage for innovative approaches to address TMAO-related health challenges in clinical settings.

### 4.5 Limitations and prospects

Although this MR study provides genetic evidence of a causal relationship between the gut microbiome and TMAO, it has some limitations. Both the gut microbiome and TMAO datasets used in this study were from Europe, thus potentially limiting the generalizability of our findings to other ethnicities. Additionally, although this study revealed the significant causal effects of six gut microbes on TMAO, the biological mechanisms by which they modulate it remain unclear. There may also be unrecognized confounding variables between the gut microbiome and TMAO, which increases the potential risk of bias. Given these limitations, future studies should expand the datasets of the gut microbiome and TMAO to other races, aiming for multiracial MR analysis and equitable health outcomes. We expect more detailed and comprehensive cohort studies in the future to validate the relationship between these six gut microbes and TMAO. Finally, we anticipate that further animal experiments will explore the mechanisms through which these gut microbes modulate TMAO, thereby offering objective biological insights.

## 5 Conclusion

Our findings suggest that the *phylum Pseudomonadota, family Sutterellaceae*, and *species Bacteroides finegoldii*, as well as *Bacteroides uniformis* are associated with increased TMAO. In contrast, the *species Bilophila wadsworthia* and *Bacteroides thetaiotaomicron* are associated with reduced TMAO. This study provides novel insights into the relationship between the gut microbiome and TMAO levels. However, owing to the current scarcity of evidence, further biological studies are necessary to explore the mechanisms by which these gut microbes regulate TMAO.

## Data Availability

The original contributions presented in the study are included in the article/Supplementary material, further inquiries can be directed to the corresponding author.
